# Lipid Profile, Antioxidant and Antihypertensive Activity, and Computational Molecular Docking of Diatom Fatty Acids as ACE Inhibitors

**DOI:** 10.3390/antiox11020186

**Published:** 2022-01-19

**Authors:** Jeeraporn Pekkoh, Kittiya Phinyo, Theera Thurakit, Sureeporn Lomakool, Kritsana Duangjan, Khomsan Ruangrit, Chayakorn Pumas, Supat Jiranusornkul, Wipawadee Yooin, Benjamas Cheirsilp, Wasu Pathom-aree, Sirasit Srinuanpan

**Affiliations:** 1 Department of Biology, Faculty of Science, Chiang Mai University, Chiang Mai 50200, Thailand; jeeraporn.p@cmu.ac.th (J.P.); phinyo.sc@gmail.com (K.P.); thurakit.t@gmail.com (T.T.); lomakool@gmail.com (S.L.); chayakorn.pumas@gmail.com (C.P.); wasu215793@gmail.com (W.P.-a.); 2 Science and Technology Research Institute, Chiang Mai University, Chiang Mai 50200, Thailand; kritsana.du@gmail.com (K.D.); khomsan.ruang@cmu.ac.th (K.R.); 3 Research Center of Microbial Diversity and Sustainable Utilization, Faculty of Science, Chiang Mai University, Chiang Mai 50200, Thailand; 4 Laboratory for Molecular Design and Simulation (LMDS), Faculty of Pharmacy, Chiang Mai University, Chiang Mai 50200, Thailand; supat.jira@cmu.ac.th; 5 Department of Pharmaceutical Sciences, Faculty of Pharmacy, Chiang Mai University, Chiang Mai 50200, Thailand; 6 Cluster of Excellence on Biodiversity-Based Economic and Society (B.BES-CMU), Chiang Mai University, Chiang Mai 50200, Thailand; 7 Center of Excellence in Innovative Biotechnology for Sustainable Utilization of Bioresources, Faculty of Agro-Industry, Prince of Songkla University, Songkhla 90110, Thailand; benjamas.che@psu.ac.th

**Keywords:** fatty acids, antioxidant, antihypertensive, nutritional quality indices, molecular docking, microalgal diatom

## Abstract

Diatoms, as single cell eukaryotic microalgae, are rich sources of lipids, which have either beneficial or detrimental effects on the prevention and treatment of many diseases. Gas chromatography-mass spectrometry (GC-MS) identified diatom lipids with high levels of essential fatty acids (EFAs), especially polyunsaturated FAs (PUFAs) containing both omega-3 and omega-6. Nutritional values of FAs indicated possible applications in the pharmaceutical, nutraceutical, and functional food industries. Diatom FAs showed antioxidative potential on harmful radicals by 2,2-diphenyl-1-picrylhydrazyl (DPPH) and 2,2’-azino-bis (3-ethylbenzthiazoline-6-sulphonic acid) (ABTS) scavenging, with high inhibition of the angiotensin-converting enzyme (ACE) that causes cardiovascular disease (CVD) and hypertension. A computational molecular docking simulation confirmed the inhibition mechanisms of FAs on ACE, with comparable levels of binding free energy to chemically synthesized ACE drugs. Findings suggested that diatom lipids showed potential for use as alternative ACE inhibitors or food supplement for CVD prevention.

## 1. Introduction

Microalgae emerged as a significant source of foods, chemicals, and energy due to the presence of a variety of chemical compounds that accumulate in their cells [1]. Many microalgal species such as *Chlorella vulgaris*, *Spirulina (Arthrospira) platensis,* and *Haematococcus pluvialis* can be cultivated on a massive scale and grow quickly under photosynthetic conditions [2], with also the added benefit of remediating wastewater from certain industrial facilities and animal operations [3]. Diatoms are one of the most important microalgae and extensively dispersed in the world’s seas, accounting for 20–25% of total global carbon fixation [4]. Diatom skeletons are composed of a naturally occurring material known as silica, a key component of diatomaceous earth that has many industrial uses [4]. Diatoms show great potential as primary contributors to the success of alternative renewable feedstocks, while many chemical production processes based on diatom biomass have focused on lipids as a feedstock for biodiesel production [5]. Many studies investigated the lipid production of various diatom species such as *Amphora copulate*, *Chaetoceros gracilis*, *Chaetoceros curvisetus,* and *Thalassiosira weissflogii*, with results ranging from 18−70% weight-averaged [3]. Fatty acid compositions of diatom lipids are comparable to those found in plant oils and other microalgae and are mostly composed of long-chain fatty acids [5]. Hence, diatom microalgae have recently attracted attention as potential sources of lipids for third-generation biofuels.

One intriguing aspect is the enhancing value potential of diatom lipids to include bioactive chemicals that provide new sources of bioactive molecules for use in nutraceutical and pharmaceutical applications. Diatom lipids comprise a varied group of fatty acids including saturated fatty acids (SFAs), monounsaturated fatty acids (MUFAs), and polyunsaturated fatty acids (PUFAs) that play significant roles in human metabolism, health and illness [6]. PUFAs have numerous benefits, particularly the omega-3 group. This includes eicosapentaenoic acid (EPA) and docosahexaenoic acid (DHA) that lower oxidative stress and blood pressure, while also improving immune response and insulin resistance. They are also effective in the prevention of heart disease and other chronic diseases [7]. Another category of beneficial fatty acids is omega-6, which includes linoleic acid (LA), gamma-linolenic acid (GLA), arachidonic acid (AA) and dihomo-gamma-linolenic acid (DGLA). These fatty acids alleviate the symptoms of inflammatory diseases such as arthritis [8]. However, dietary intake of diatom biomass is not recommended for human nutrition due to the presence of silica cell walls in diatoms, which may have negative effects on human health. Therefore, this study investigated the use of diatom biomass as raw lipid extracts for the purpose of harvesting rich sources of bioactive fatty acids.

Preliminary evaluations of the potential use of fatty acids in food, nutraceuticals, and pharmaceutical applications assessed nutritional quality indices such as PUFAs to SFAs ratio, index of atherogenicity, index of thrombogenicity, hypocholesterolemic to hypercholesterolemic ratio, health-promoting index, unsaturation index, sum of EPA and DHA, and *trans* fatty acid [9]. Nutritional quality indicators were developed based on the fatty acid content of foods or products, and were extensively documented in the nutritional quality of marine animal products [9,10]. The most crucial step in estimating the nutritional and/or medicinal value of fatty acids is the rapid determination of their composition. Most previous research employed a gas chromatography-flame ionization detector (GC-FID); however, nowadays, gas chromatography-mass spectrometry (GC-MS) is regarded as a more powerful and accurate approach for fatty acid detection [6]. Hence, here, the fatty acid profiles of diatom lipid extracts, especially those containing PUFAs, were characterized using a GC-MS approach.

Antioxidant fatty acids derived from microalgae have the potential to protect cells from oxidative stress by scavenging reactive oxygen species (ROS) or free radicals (e.g., 2,2-diphenyl-1-picrylhydrazyl (DPPH) and 2,2′-azinobis-(3-ethylbenzthiazolin-6-sulfonic acid (ABTS)) [6]. Fatty acids neutralize free radicals through interaction with oxygen molecules during the destabilization process and prevent them from causing damage. The buildup of ROS causes overexpression of the angiotensin-converting enzyme (ACE), which is a significant risk factor for cardiovascular disease (CVD) and hypertension. People experiencing symptoms have blood arteries that are under continuous pressure due to high blood pressure [11]. ACE, which is produced by endothelial cells and is a major player in the renin-angiotensin system, plays a significant role in the development of cardiovascular disease by controlling blood pressure and water balance in the body [12]. Hence, ROS scavengers and ACE inhibitors slow down the production of ROS and suppress the expression of ACE. Previous studies proved that fatty acids derived from microalgal biomass inhibited both ROS production [13] and ACE activity [14]. However, few reports are available on the molecular mechanisms of action between diatom fatty acids and ACE.

Therefore, this study evaluated the bioactivities of lipid extracts including *Anomoeoneis* sp. AARL D039 and *Rhopalodia* sp. AARL D020 derived from diatom biomass. Lipid yields and fatty acid composition were analyzed using GC-MS and nutritional quality indices of the obtained lipid extracts were also evaluated. Antioxidant properties including radical scavenging activities of ABTS and DPPH and the inhibition effect on ACE were determined. To establish the mechanism of inhibition, the molecular docking approach was performed to explain the binding interaction between diatom fatty acids and ACE.

## 2. Materials and Methods

### 2.1. Diatom Samples

Two diatom microalgae, *Anomoeoneis* sp. AARL D039 (Ano) and *Rhopalodia* sp. AARL D020 (Rho), obtained from the Applied Algal Research and Laboratory (AARL), Faculty of Science, Chiang Mai University, Thailand were cultivated in 20 L of Bold’s Basal Medium under 25 °C with continuous light feeding at 80 μmol/s/m^2^. After 30 days of cultivation, the microalgal biomass was harvested using centrifugation at 4226× *g* for 15 min. The pellets were washed twice with distilled water and then dried at 60 °C until constant weight.

### 2.2. Lipid Extraction

Lipid extraction was carried out following the modified methods of Conde et al. [6] and Vilakazi et al. [13]. Dried diatom biomass (5.0 g) was mixed with 50 mL of solvent mixture (dichloromethane/methanol = 1/1 *v*/*v*). The mixtures were subjected to cell disruption using sonication at 40% amplitude for 30 min and extracted twice. After that, 50 mL of dichloromethane and 25 mL of distilled water were added to the mixture, resulting in two layers. The mixture was centrifuged at 4226× *g* for 5 min. The dichloromethane layer was collected and filtered with 0.2 µm nylon membrane filters. The extracted lipids were centrifuged at 4226× *g* for 5 min to obtain a clear supernatant, and the impurities such as tocopherols, carotenoids and other pigments were completely removed using activated carbon and the solvent was removed by feeding a nitrogen gas stream. The extracted lipid was dried and weighed, with lipid content calculated as the percentage of lipid to dried biomass.

### 2.3. Analysis of Fatty Acid Composition

The extracted lipid was converted to fatty acid methyl esters (FAMEs) by acid-catalyzed transesterification [15]. The extracted lipid sample (10 mg) was mixed with 0.5 mL toluene, 1.5 mL of methanol and 50 μL of 35% HCl and the mixtures were incubated at 98 °C for 2 h. After cooling, 1 mL of hexane was added and vortexed. The hexane layer (FAME) was collected before analyzing the FAME composition using a 7890B Gas Chromatograph equipped with a cross-linked capillary HP-5 column (length 30 m, 0.32 mm I.D, 0.25 μm film thickness). The GC equipment was connected to a 5975C inert XL EI/CI MSD with a Triple-Axis Detector operated at 70 eV and a scanning range of *m*/*z* 50–550 (1 s cycle in a full scan mode). Operating conditions were as follows: inlet temperature 240 °C, initial oven temperature 45 °C held for 2 min, then ramped to 100 °C at 25 °C/min, held for 10 min, then ramped to 190 °C at 5 °C/min, held for 10 min, then ramped to 220 °C at 5 °C/min, held for 6 min. Finally, the post run was adjusted at 250 °C for 1 min with detector temperature at 280 °C. Identification of the FAMEs was carried out by the retention time and comparison of the MS spectrum with the Wiley W10N11 and NIST chemical database library and confirmed using the 37 component FAME mixed standard.

### 2.4. Evaluation of Nutritional Quality Indices

Fatty acid profiling was used to calculate the nutritional indices of diatom lipids such as polyunsaturated fatty acid (PUFA)/saturated fatty acid (SFA) ratio (PS), index of atherogenicity (IA), index of thrombogenicity (IT), hypocholesterolemic/hypercholesterolemic ratio (h/H), health-promoting index (HPI), unsaturation index (UI), sum of eicosapentaenoic acid and docosahexaenoic acid (SED), and trans fatty acid (TFA) using empirical Equations (1)–(8), respectively, as reported by Chen and Liu [9].

PS = PUFA/SFA (1)

IA = [C12:0 + (4 × C14:0) + C16:0]/ΣUFA(2)

IT = (C14:0 + C16:0 + C18:0)/[(0.5 × ΣMUFA) + (0.5 × Σn − 6 PUFA) + (3 × Σn − 3 PUFA) + (n − 3/n − 6)]                      (3)

h/H = (cis-C18:1 + ΣPUFA)/(C12:0 + C14:0 + C16:0)(4)

HPI = ΣUFA/[C12:0 + (4 × C14:0) + C16:0](5)

UI = [1 × (%monoenoics)] + [2 × (%dienoics)] + [3 × (%trienoics)] + [4 × (%tetraenoics)] + [5 × (%pentaenoics)] + [6 × (%hexaenoics)](6)

SED = EPA + DHA(7)

TFA = ΣTFA(8)

### 2.5. Bioactivities of Lipid Extract

#### Determination of Antioxidant Properties

- DPPH radical scavenging activity

A 50 µL of sample (0.5–5.0 mg/mL) or Trolox as a standard compound (1–10 µg/mL) in methanol was added into a 96-well plate that contained 150 µL of 0.16 mM DPPH methanolic solution. The mixture was left to stand at room temperature for 30 min in the dark, and its absorbance was detected at 517 nm [16]. The ability to inhibit the DPPH radical was calculated using equation (Equation (9)):

DPPH radical scavenging activity (%) = [(A_c_ − (A_s_ − A_sc_))/A_c_] × 100 (9)

where A_c_ is the control absorbance as the DPPH solution without sample, A_s_ is the absorbance of the test sample (DPPH solution plus test sample) and A_sc_ is the absorbance of the sample only (sample without DPPH solution).

- ABTS radical scavenging activity

The stock solution of ABTS^·+^ radical was prepared by mixing 7 mM ABTS in water with 2.45 mM potassium persulfate at 1:1 *v*/*v* and kept in the dark at room temperature for 12–16 h before use. The ABTS^·+^ solution was then diluted with methanol to obtain an absorbance of 0.70 ± 0.02 at 734 nm [2]. The reaction was engendered in a 96-well plate composed of 195 µL ABTS^·+^ solution and 5 µL of sample (0.5–10 mg/mL) or Trolox (0.05–0.5 mg/mL). The mixture was gently shaken and left to stand at 37 °C for 10 min. The absorbance was read at 734 nm. The ability to inhibit the ABTS^·+^ radical was calculated using equation (Equation (10)):

ABTS^·+^ radical scavenging activity (%) = [(A_c_ − (A_s_ − A_sc_))/A_c_] × 100 (10)

where A_c_ is the control absorbance as the ABTS^·+^ solution without sample, A_s_ is the absorbance of the test sample (ABTS^·+^ solution plus test sample) and A_sc_ is the absorbance of the sample only (sample without ABTS^·+^ solution).

- Angiotensin-converting enzyme (ACE) inhibitory activity

A 5 µL ACE solution (200 mU/mL) was added to 31 µL of 50 mM sodium borate buffer pH 8.3 containing 0.3 M NaCl (SBBS) in each well of a 96-well plate. A 10 µL sample or SBBS (control; C) reaction was added. Enalapril (2–12 µg/mL) was used as a standard compound. The reaction was started by the addition of 13 µL of substrate HHL solution (5 mM) to the reaction mixture (final volume of 59 µL). Two blanks were prepared: one without ACE and inhibitor peptide (Bi) and another without ACE and HHL (Bs). After incubation for 1 h at 37 °C, 100 µL sodium tetraborate (200 mM), 50 µL sodium sulfite (10 mM) and 50 µL TNBS (3.4 mM) were added to each well. The mixtures were further incubated for 20 min at 37 °C. The absorbance was measured at 420 nm and used for calculating the percentage of ACE inhibitory activity according to the following equation (Equation (11)) [11]:

ACE inhibitory activity (%) = [(C − Bi) − (S − Bs)/(C − Bi)] × 100(11)

### 2.6. Molecular Docking Analysis of ACE

#### 2.6.1. Preparation of Target Protein and Ligands

Angiotensin-converting enzyme (ACE) is a zinc metallopeptidase with two domains as N-domain (nACE) and C-domain (cACE). ACE is involved in the regulation of blood pressure by hydrolyzing angiotensin I to produce angiotensin II. Both ACE domains have a similar sequence and an elliptical 3D structure, where the binding cavity is divided into four subsites; S2, S1, S1′, and S2′, and contains a catalytic zinc ion (Zn^2+^) in the middle between S1 and S1′ [17]. The two domains are structurally similar, but they have different pharmacological effects. C-domain is a major domain that produces angiotensin II and regulates blood pressure [18]. The 3D X-ray crystal structure of human cACE (PDB ID: 6H5W) with an inhibitor omapatrilat was retrieved from the RCSB Protein Data Bank [19]. Human cACE was prepared using the Discovery Studio 2.5 software (Discovery, New York, NY, USA) to identify the mechanism of the ligand-receptor interaction. Omapatrilat and all hetero atoms were removed except for Zn^2+^, an atom influenced by the interaction of approved ACE inhibitors [17]. The cACE structure was then cleaned and the hydrogen atoms were added.

For the ligands, enalaprilat, the active form of enalapril as an approved ACE inhibitor was chosen as a positive control. Three-dimensional structures of all fatty acids and enalaprilat were obtained from the available structures on the PubChem compound database. The 3D structures were unavailable for steric acid, eicosadienoic acid, erucic acid, behenic acid, nervonic acid, and lignoceric acid, and 2D structures were downloaded instead and converted into 3D structures using Discovery Studio 2.5 software. The ionization behavior of carboxylic acids of all fatty acids (FAs) at a physiological pH of 7.4 was set [20]. The pKa values obtained from the PubChem compound database showed that all FAs were ionized at almost 100%, implying that the hydrogen atom of the carboxylic group was removed from the structures. These 3D structures were then optimized by the Gaussian 09 program (Gaussian Inc., Wallingford, CT, USA) using B3LYP model with a 6–31G (d, p) basis set [21].

#### 2.6.2. Molecular Docking Analysis

All fatty acids and enalaprilat, a positive control, were docked into the cACE binding site using AutoDock 4.2.6 and AutoDockTools as the graphical user interface. For all the receptors for all dockings, a cubic grid box (40 × 40 × 40 Å) was generated with the center at 13.162, −6.382, 20.700 (XYZ coordinate), close to Zn^2+^, and the grid spacing was kept to 0.375 Å. For each of the ligands, 100 poses were generated using high-accuracy Lamarckian genetic algorithm searches with an initial population size of 300 random positions and conformations. Each run had two stop criteria: a maximum of 2,500,000 energy evaluations or a maximum of 27,000 generations. Docked poses were clustered by the reference root-mean-square deviation (RMSD) tolerance of 2 Å and ranked according to their binding energies. The docked conformation with the lowest binding energy of the most populated cluster was selected for interaction and binding mode analysis using Discovery Studio 2.5 and Pymol software (Schrödinger, Inc., New York, NY, USA).

### 2.7. Statistical Analysis

Triplicate analyses were performed, with results subjected to descriptive statistical analysis using one-way ANOVA (analysis of variance) and Duncan’s multiple range tests (*p* < 0.05) using SPSS statistics 17.0.

## 3. Results and Discussion

### 3.1. Characterization of Diatom Lipids

In this study, two microalgal diatom strains as *Anomoeoneis* sp. AARL D039 (Ano) and *Rhopalodia* sp. AARL D020 (Rho) were used to extract lipids using dichloromethane/methanol as the solvent. The lipid content of diatom Rho (12.72% *w*/*w*) was higher than diatom Ano (9.92% *w*/*w*) (Table 1), demonstrating the wide variation in lipid content across the different strains examined. Our results concurred with Fields and Kociolek [22] who reported lipid accumulation from 62 diatom species ranging from 6 to 66% *w/w*. Variations in findings were attributable to the different growing conditions of microalgae [6], while additional molecules other than lipids from the solvent employed in the extraction process contributed to the total weight of lipid [23].

Fatty acids (FAs) in diatom lipids of Ano and Rho were identified after transesterification reactions and quantified as fatty acid methyl esters (FAMEs) using gas chromatography-mass spectrophotometry (GC-MS). The FAs presented in this study were esterified and regarded as more bioavailable than their free forms [6,24]. The GC-MS results indicated that both diatom FAs included saturated fatty acids (SFAs, 41.41–41.75%), monounsaturated fatty acids (MUFAs, 35.53–42.71%) and polyunsaturated fatty acids (PUFAs, 15.54–23.06%). The main FAs in Ano lipid were palmitoleic acid (C16:1, 34.61%), followed by palmitic acid (C16:0, 33.02%) and arachidonic acid (C20:4n6, 10.27%), while Rho lipid showed high palmitic acid (C16:0, 31.22%) levels followed by palmitoleic acid (C16:1, 24.67%) and linoleic acid (C18:2n6c, 12.58%) (Table 1). Similar results were reported by Yi et al. [25] who recorded FAs in diatom lipids ranging from C14:0 to C22:6, and C16:0 and C16:1 as the most prevalent but with different FA contents. These discrepancies resulted from various growing conditions, different extraction procedures (e.g., employing different solvents and mixes of solvents), different derivatization methodologies and diverse data gathering systems [6].

Interestingly, both Ano and Rho contained omega-3 PUFAs and omega-6 PUFAs as essential FAs (Table 1). Overall amounts of omega-3 PUFAs (2.28–2.81%) were lower than total levels of omega-6 PUFAs (13.26–20.25%). FAs rich in PUFAs, particularly omega-3 PUFAs have beneficial effects on health, lowering the risk of cardiovascular disease and atherosclerosis, as well as cancer and inflammatory illnesses [26,27]. Omega-6 PUFAs are also beneficial for humans and mammals in very small doses since they serve as precursors of omega-3 PUFAs [27]. The lower omega-3 PUFAs are caused by low expression of FA biosynthesis-related genes, especially the omega-3 fatty acid desaturase (ω-3 FAD) gene [28].

Omega-3 PUFAs contained eicosapentaenoic acid (EPA; C20:5n3, 1.33% for Ano and 2.61% for Rho) (Table 1). and docosahexaenoic acid (DHA; C22:6n3, 0.95 for Ano and 0.20% for Rho) (Table 1). Both diatom lipids contained lower quantities of EPA and DHA that can be increased by modifying growth parameters such as salinity, temperature, light intensity and nutrients [13]. This should be examined in further studies. Omega-6 PUFAs accounted for 20.25% of the total PUFA content in Rho FAs with appreciable levels of linoleic acid (C18:2n6c, 12.58%) and arachidonic acid (ARA; C20:4n6, 5.26%), while omega-6 PUFAs of Ano FAs had the highest levels of ARA at 10.27%. Similar observations on omega-6 PUFA composition in diatom FAs were reported at different concentrations by Yi et al. [25] and Blasio and Balzano [27].

PUFAs with high concentrations of EPA, DHA, and ARA are regarded as good candidates for prospective uses in pharmaceuticals, cosmetics and dietary supplements [25]. Yi et al. [25] and Blasio and Balzano [27] reported that PUFAs can be intracellularly biosynthesized by humans and other mammals from SFAs and MUFAs synthesized from carbon groups in carbohydrates and proteins through a series of desaturation (addition of a double bond) and elongation reactions; however, the conversion efficiency is insufficient to insert a *cis* double bond into the n − 6 or n − 3 position of FA, resulting in lower levels of intracellular PUFAs, especially EPA and DHA. The consumption of PUFA-rich foods is also strongly advocated. Fatty acid profiling results suggested that both Ano and Rho diatom FAs showed potential for use as bioactive ingredients for dietary supplements with high nutritional qualities.

### 3.2. Nutritional Quality Indices

FAs from food sources each have a distinct composition that influences health outcomes positively or negatively [9]. Thus, their nutritional and/or medicinal values must be determined. In this study, the nutritional indices of diatom FAs, such as PUFA/SFA ratio (PS), index of atherogenicity (IA), index of thrombogenicity (IT), hypocholesterolemic to hypercholesterolemic ratio (h/H), health-promoting index (HPI), unsaturation index (UI), sum of eicosapentaenoic acid and docosahexaenoic acid (SED), and total trans fatty acid (TFA) were calculated based on the fatty acid composition, as summarized in Table 2. The PUFA/SFA ratio (PS) was 0.37 for Ano and 0.56 for Rho, concurring with Maneechote et al. [1] for microalgal lipids (0.26–0.87) and Aliyu et al. [29] for plant oils (0.03–1.54), indicating their nutritional quality. The PS nutrient indicator is often used to evaluate the influence of diet on cardiovascular health, with higher PS levels resulting in a more positive effect [9].

The index of atherogenicity (IA) indicates the degree of cardiovascular disease (CVD) risk. Yurchenko et al. [30] suggested that eating foods or products with lower IA values reduced total cholesterol and LDL-cholesterol levels in human blood plasma. The IA was 0.68 for Ano and 0.94 for Rho (Table 2) corresponding to the regular rate of IA with values 0.21 to 1.41 for fish [9] and 0.49 to 1.70 for microalgae [31]. The risk of blood clots forming in blood arteries is assessed using the index of thrombogenicity (IT). IT was used as a nutritional index to assess the beneficial effect of seaweed, fish and even microalgae, giving IT levels of 0.49 to 1.70 [9,31]. The IT values of both Ano and Rho were comparable, ranging 1.11 to 1.15 (Table 2). Chen and Liu [9] suggested that foods or products with lower IT levels were beneficial for the prevention of CVD. Results indicated that both Ano and Rho FAs with appreciable levels of IA had high nutritional quality and could be used as bioactive supplements to reduce the risk of CVD.

The hypocholesterolemic to hypercholesterolemic ratio (h/H) is a nutritional indicator that was developed to evaluate the impact of FAs on cholesterol levels in humans. Higher h/H combined with lower IA and IT, as shown by Chen and Liu [9], contributed to a drop in cholesterol levels, ultimately leading to a reduction in coronary heart disease (CHD). In this study, the h/H was 0.57 for Ano and 0.82 for Rho, and in line with previously reported h/H values obtained from algae, shellfish, fish, meat, and dairy products ranging 0.21 to 2.78 [9,32]. The health-promoting index (HPI) was 1.46 for Ano and 1.06 for Rho (Table 2), corresponding to Chen and Liu [9]. Generally, HPI is mostly employed in dairy product studies, such as milk and cheese, where HPI values ranged 0.16 to 0.66. According to Chen and Liu [9], products having high HPI values are more beneficial for human health than those with lower HPI values. Therefore, both Ano and Rho FAs suggested improved health-promoting potential.

The unsaturation index (UI) was 104.01 for Ano and 102.72 for Rho (Table 2). Chen and Liu [9] reported that UI values of seaweed, microalgae, crops, and meat ranged 45 to 369. The UI is regularly used as a criterion to estimate the composition of macroalgal FAs to determine the amount of high-quality PUFAs. Microalgae can be employed as an alternative source of high-quality PUFAs rather than fish or seafood oil. EPA and DHA are omega-3 PUFAs that are required for the proper functioning of the human body’s biological systems, with the potential to lower the risk of cardiovascular disease, hypertension, and inflammation. The sum of EPA and DHA (SED) is an essential nutritional indicator for foods or products, especially seafood and seafood products [32]. The SED values for Ano and Rho FF were 2.28% and 2.81%, respectively, and in line with previously reported SED values of microalgae ranging 0.2 to 50% [1,33]. These findings suggested that the presence of EPA and DHA in both Ano and Rho FAs provided valuable nutritional quality for human consumption.

The *trans* fatty acids (TFAs), such as those found in *trans*-MUFAs and *trans*-PUFAs must also be considered when assessing the safety of foods or products since they have a detrimental impact on a variety of vital human bodily functions. TFAs consumed should be less than 1% of total calorie consumption according to World Health Organization (WHO)/Food and Agriculture Organization (FAO) recommendations. The 2020–2025 Dietary Guidelines for Americans suggested that people should minimize their intake of TFAs by reducing foods that included artificial sources of *trans* fats, while the Food Standards Agency of the United Kingdom recommended daily consumption of TFA at less than 2% of total daily energy, or 5 g/day [9]. A few clinical experiments with normotensive participants showed that TFAs derived from hydrogenated oil had no impact on either systolic or diastolic blood pressure [34]. Raff et al. [35] found that daily consumption of 3.6 g of TFAs derived from milk fat for five weeks had no effect on blood pressure or isobaric arterial elasticity. In this study, the TFA contents of Ano and Rho were 2.26% and 3.14% (Table 2). Similar TFA values were observed in edible seaweed, plant oil and microalgae by Maneechote et al. [1], Aliyu et al. [29] and Matos et al. [31], with values ranging from 1% to 10%.

The several nutritional index evaluations employed in this study indicated that both diatom Ano and Rho FAs had good nutritional quality, with potential use in nutraceutical and pharmaceutical applications to prevent or reduce the risk of severe diseases.

### 3.3. Antioxidant Properties

**Figure 1:**
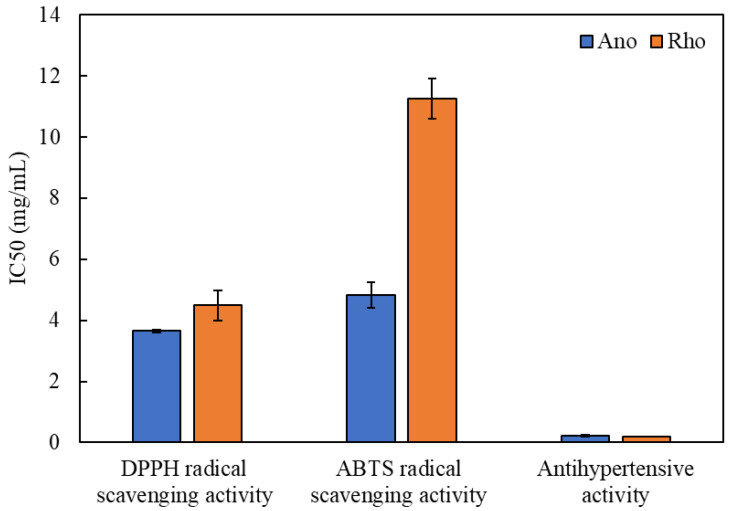
Antioxidant properties and antihypertensive activity of two diatom lipids (Rho; *Rhopalodia* sp. AARL D020 and Ano; *Anomoeoneis* sp. AARL D039).

Reactive oxygen species (ROS) or free radical secretion results in the occurrence of oxidative stress in mammalian cells, which leads to the development of a variety of illness disorders [16]. The DPPH and ABTS free radicals are hazardous molecules and detrimental to human health, causing disorders such as cancer and hypertension [11]. The DPPH and ABTS radical scavenging assays are widely used to access the biological potentials of natural extracts due to their methodological simplicity and stability. Here, both assays were used to evaluate the antioxidant properties of the two diatom lipids, with Ano showing greater half-maximal inhibitory concentration (IC50) than Rho (Figure 1). Lower IC50 values indicated that the extracts showed potential for use in pharmaceutical, nutraceutical and cosmetic applications [16]. Ano gave IC50 values for scavenging DPPH and ABTS free radicals at 3.65 mg/mL and 4.83 mg/mL, respectively, while IC50 values for DPPH and ABTS radical scavenging activity of Rho were 4.49 mg/mL and 11.26 mg/mL, respectively (Figure 1). When compared to the antioxidant activity of Trolox, a reference compound with the IC50 values of 7.68 µg/mL and 0.30 mg/mL for scavenging DPPH and ABTS free radicals, respectively, the antioxidant activity of FAs was found to be lower than that of Trolox. This might be because Trolox is a single compound acting as radical scavengers, while FAs contained both active and inactive forms. However, our results agreed with previous studies on algal lipids obtained from *Grateloupia turuturu*, *Schizochytrium* sp., *Nannochloropsis oceanica, Chlorella vulgaris,* and *Acutodesmus obliquus* with IC50 for DPPH scavenging assays of 0.2 to 30 mg/mL [36,37], and ABTS IC50 values of 0.1 to 80 mg/mL [6,36,38]. Results indicated that the lipid extracts of Ano and Rho had reducing ability to stabilize unstable DPPH free radicals by converting them to a more stable form [15] and reducing the ABTS free radical through the oxidation reaction [6].

Banskota et al. [23] found that the antioxidant activity of microalgal lipid extracts was enhanced by the presence of lipids with greater levels of UFAs, especially PUFAs. PUFAs were linked to antioxidant activity in lipid extracts, but the proportion of omega-3 or omega-6 PUFAs in the lipid extract did not influence this activity [6]. Our results were inconsistent with these reports, probably due to FA composition and concentration variations between the microalgae species used. When compared to Ano, Rho had high concentration of PUFAs (Table 1) but low antioxidant properties in both ABTS and DPPH assays (Figure 1). This indicated that antioxidant potential in our study correlated with the amount of MUFAs, resulting in higher scavenging activity of both DPPH and ABTS free radicals by Ano (Table 1 and Figure 1). Overall results suggested that both Ano and Rho lipids could be used as a supplement or directly as food due to their bioactive potentials.

### 3.4. Antihypertensive Activity

The most significant risk factor for cardiovascular disease is high blood pressure (hypertension). People displaying symptoms have blood vessels that are impacted by continual high pressure [7,26]. The angiotensin-I-converting enzyme (ACE) is critical in actively controlling blood pressure, and treatments for hypertension include blocking this specific enzyme [11]. In this study, the inhibition effects of Ano and Rho lipids having beneficial FAs on ACE were evaluated and expressed as the IC50 values of antihypertensive activity or ACE inhibitory activity. The ACE inhibitory activities of both diatom lipid extracts were comparable, with IC50 values of 0.22 mg/mL for Ano and 0.20 mg/mL for Rho (Figure 1), but lower than those found in enalapril as a standard compound (3.6 µg/mL). Despite FAs having lower ACE inhibition than that of enalapril, FAs might be employed as alternative ACE inhibitors to prevent the negative effects of enalapril and other commercially related medicines [11,12]. It was possible that SFAs, MUFAs, and PUFAs in diatom lipids behaved as ACE inhibitors or cardioprotective molecules. Similar observations were documented by Kim et al. [39] who found that both SFAs and UFAs from *Semisulcospira coreana* lipid extracts were involved in ACE inhibitory activities with IC50 values of 0.1 to 0.5 mg/mL. In the literature, the ability of FAs to inhibit ACE activity correlated with high levels of MUFAs and PUFAs such as EPA, DHA, ARA, GLA (gamma-linolenic acid), and LA (linoleic acid) [7]. Das et al. [26] also discovered that long-chain PUFAs inhibited ACE activity, decreased angiotensin II formation, increased NO generation and suppressed TGF-expression, and were beneficial for the prevention of primary hypertension in humans. The availability of appropriate levels of long-chain PUFAs during key stages of teenage growth was suggested to reduce the development of hypertension in adult life.

In the human body, ACE functions as the primary enzyme in the Renin-Angiotensin System (RAS) pathway, catalyzing the conversion of Angiotensin I (Ang I; inactive form) to the potent vasoconstrictor Angiotensin II (Ang II; highly active form). ACE also destroys bradykinin, which has a vasodilatory effect. Bradykininogen is the source of active hypertensive bradykinin, which is subsequently broken down into inactive parts as a result of the actions of kinase II [12]. Therefore, FAs in Abo and Rho lipids have the potential to react with ACE, rendering it inaccessible for the conversion of Ang I to Ang II in the human body, resulting in decreased Ang II formation and reduced bradykinin degradation. Our results suggested that diatom FAs could be used as alternative ACE inhibitors or antihypertensive drugs, as well as food supplements to avoid reported side-effects associated with synthetic drugs, such as rashes on the skin, coughing, and disruptions to the sense of taste [11,12].

### 3.5. Computational Molecular Docking Simulation

To understand the molecular mechanisms of 24 FAs, derived from *Anomoeoneis* sp. AARL D039 (Ano) and *Rhopalodia* sp. AARL D020 (Rho), in inhibiting the action of ACE, molecular docking studies were used to illustrate molecular interactions and binding affinities of FAs in binding sites of ACE. Values of binding free energies calculated by AutoDock of enalaprilat and FAs are reported in Table 3. Higher negative binding free energy indicates a greater binding affinity with the receptor. Enalaprilat showed the strongest binding interaction with binding free energy at −14.15 kcal/mol, while, *cis*-4,7,10,13,16,19-docosahexaenoic acid (docosahexaenoic acid, DHA), *cis*-5,8,11,14,17-eicosapentaenoic acid (eicosapentaenoic acid, EPA) and *cis*-5,8,11,14-eicosatetraenoic acid (arachidonic acid) were the top three active FA ingredients for cACE inhibition. The AutoDock binding free energy showed that other FAs were also able to bind to the binding site of cACE and tended to bind better with increased carbon chain length and the increased *cis* position.

**Figure 2:**
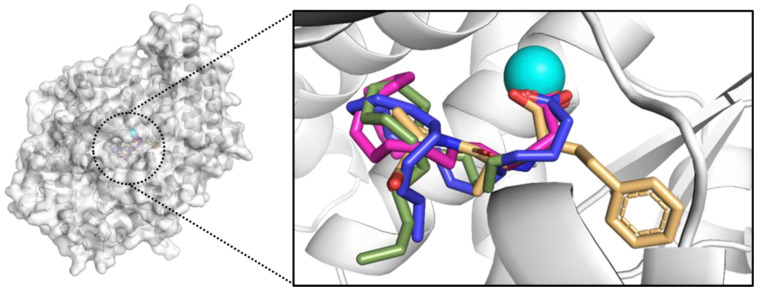
Superimposing model of enalaprilat (light orange) and top three best fatty acids (docosahexaenoic acid: DHA, blue; eicosapentaenoic acid: EPA, magenta; arachidonic acid: ARA, green) on angiotensin-converting enzyme (ACE) binding.

**Figure 3:**
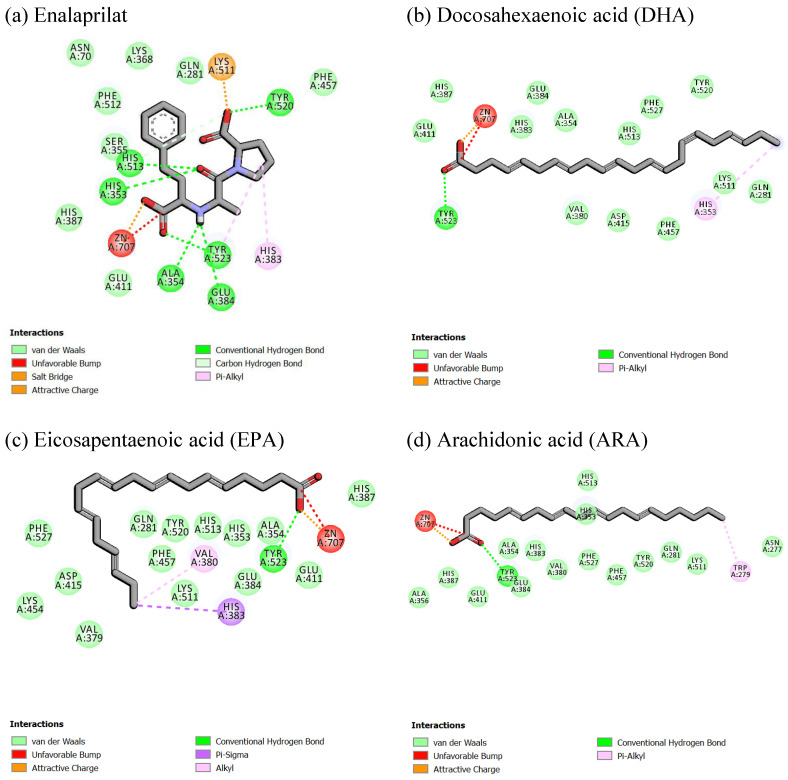
2D interactions of enalaprilat and top three best fatty acids (docosahexaenoic acid: DHA; eicosapentaenoic acid: EPA; arachidonic acid: ARA) on angiotensin-converting enzyme (ACE) binding.

Molecular interaction and binding mode conformations of key residues were analyzed using Discovery Studio 2.5 and Pymol software. The top three FAs and enalaprilat superimposed in the binding site of cACE are indicated in Figure 2. This superimposed docking model showed that the FAs aligned in the same direction by facing the carboxylate group toward the Zn ion, similar to the arrangement of enalaprilat. The 2D interaction of enalaprilat and cACE in Figure 3 showed that one carboxylate group interacted with the zinc ion and another carboxylate group formed ionic interaction with lysine (LYS511). The amino-terminal phenyl moiety of enalaprilat formed van der Waals interaction with phenylalanine (PHE512), an amino acid in the S_1_ hydrophobic pocket. The central carbonyl group of enalapril formed two strong hydrogen bonds with two histidine residues (HIS353 and His 353), while the oxygen of the proline moiety carboxylate group formed a strong hydrogen bond with TYR520, and the oxygen of the amino-terminal phenyl moiety carboxylate group formed a strong hydrogen bond with TYR523. Pyrrolidine of the proline moiety also formed pi-interaction with HIS383 in S_2′_ of cACE. This information concurred with Caballero [17], demonstrating that the molecular docking protocol used in this study was validated and could be used to study other compounds for cACE binding.

Figure 2 and Figure 3 show that the studied FAs had straight-chain structures, which differed from enalaprilat, so they exhibited different interactions. No part of the FAs interacted with the S_1_ hydrophobic pocket. Interestingly, the carboxylate group of the top three FAs showed ionic interaction with the zinc ion and the hydrogen bond with TYR523, similar to enalaprilat. Carbon chains can form many van der Waals interactions with amino acids, especially amino acids in S_1′_ and S_2′_ sites. This indicated that diatom FAs played a role in ACE inhibition using a different mechanism compared to enalaprilat. Enalaprilat has a higher binding free energy than diatom FAs but there are still concerns regarding the usage of enalaprilat in clinical settings due to the possibility of adverse effects from this medication [11,12]. ACE inhibitory FA produced from naturally edible diatom microalgae did not cause any undesirable side effects and, consequently, further clinical studies are required to investigate the combination of diatom FAs with/without synthetic ACE drugs.

## 4. Conclusions

Results demonstrated that the lipid extracts derived from two microalgal diatoms (*Anomoeoneis* sp. AARL D039 and *Rhopalodia* sp. AARL D020) contained high levels of beneficial fatty acids, especially MUFAs and PUFAs, which are nutritional quality bioactive indicators. Nutritional values of the fatty acid extracts suggested possible uses for applications in the pharmaceutical, nutraceutical, and functional food industries. The fatty acids also showed potential as a future alternative to harmful free radical scavengers and ACE inhibitors due to their antioxidant and antihypertensive properties. A computational molecular docking simulation proved that the inhibition mechanisms of diatom fatty acids on ACE had comparable levels of binding free energy to that of chemically synthesized ACE drugs. Results suggested that diatom lipids with high levels of fatty acids and high nutritional values play important roles in biotechnological applications.

## Figures and Tables

**Table 1:** Fatty acid (FA) profile of *Anomoeoneis* sp. AARL D039 and *Rhopalodia* sp. AARL D020 by GC-MS.

FA	2D Structure	Concentration (%)	
		*Anomoeoneis* Sp. AARL D039	*Rhopalodia* Sp. AARL D020
Lipid Content (%*w*/*w*)		9.92 ± 1.08	12.72 ± 0.90
Saturated fatty acid (SFA)			
Caproic acid (C6:0)	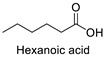	N.D.	0.07 ± 0.02
Capric acid (C10:0)	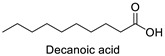	N.D.	0.07 ± 0.02
Lauric acid (C12:0)	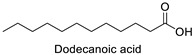	N.D.	0.12 ± 0.01
Myristic acid (C14:0)	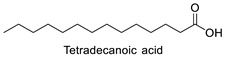	1.72 ± 0.01	5.95 ± 0.04
Pentadecanoic acid (C15:0)	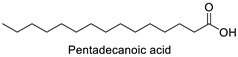	0.55 ± 0.04	0.43 ± 0.02
Palmitic acid (C16:0)	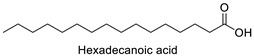	33.02 ± 0.01	31.22 ± 0.01
Margaric acid (C17:0)	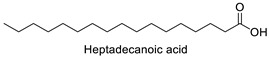	0.38 ± 0.01	0.25 ± 0.04
Steric acid (C18:0)	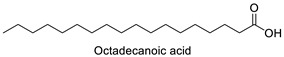	5.63 ± 0.02	3.30 ± 0.00
Behenic acid (C22:0)	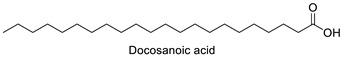	0.23 ± 0.02	N.D.
Lignoceric acid (C24:0)	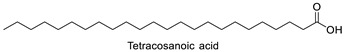	0.22 ± 0.01	N.D.
Monounsaturated fatty acids (MUFA)			
Myristoleic acid (C14:1)	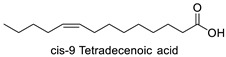	0.34 ± 0.03	0.12 ± 0.01
Palmitoleic acid (C16:1)	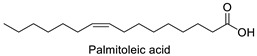	34.61 ± 0.01	24.67 ± 0.02
Ginkgolic acid (C17:1)	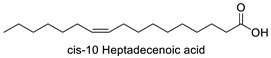	0.80 ± 0.00	0.20 ± 0.00
Elaidic acid (C18:1n9t)	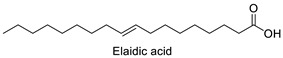	2.26 ± 0.03	3.14 ± 0.03
Oleic acid (C18:1n9c)	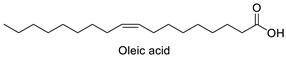	4.30 ± 0.01	7.40 ± 0.00
Erucic acid (C22:1n9)	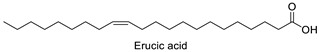	0.23 ± 0.02	N.D.
Nervonic acid (C24:1n9)	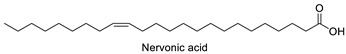	0.17 ± 0.02	N.D.
Polyunsaturated fatty acids (PUFA)			
γ-Linolenic acid (C18:3n6)	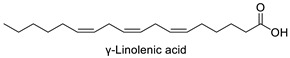	0.44 ± 0.03	1.41 ± 0.01
Linoleic acid (C18:2n6c)	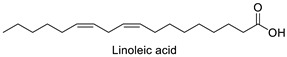	0.49 ± 0.01	12.58 ± 0.01
Arachidonic acid (C20:4n6)	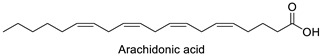	10.27 ± 0.02	5.26 ± 0.03
Eicosapentaenoic acid (C20:5n3)	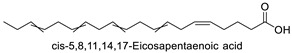	1.33 ± 0.02	2.61 ± 0.01
Dihomo-gamma-linolenic acid (C20:3n6)	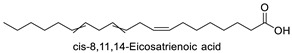	1.45 ± 0.04	0.51 ± 0.01
Eicosadienoic acid (C20:2n6)	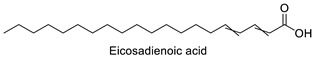	0.61 ± 0.01	0.49 ± 0.01
Docosahexaenoic acid (C22:6n3)	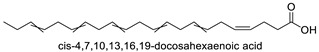	0.95 ± 0.04	0.20 ± 0.00

**Table 2:** Nutritional indexes of fatty acids derived from *Anomoeoneis* sp. AARL D039 and *Rhopalodia* sp. AARL D020.

Indexes	*Anomoeoneis* Sp. AARL D039	*Rhopalodia* Sp. AARL D020
PS	0.37 ± 0.02	0.56 ± 0.03
IA	0.68 ± 0.01	0.94 ± 0.03
IT	1.15 ± 0.03	1.11 ± 0.01
h/H	0.57 ± 0.02	0.82 ± 0.01
HPI	1.46 ± 0.02	1.06 ± 0.03
UI	104.01 ± 0.01	102.72 ± 0.20
SED	2.28 ± 0.01	2.81 ± 0.01
TFA	2.26 ± 0.03	3.14 ± 0.03

PS: polyunsaturated fatty acids/saturated fatty acids ratio; IA: index of atherogenicity; IT: index of thrombogenicity; h/H: hypocholesterolemic/hypercholesterolemic ratio; HPI: health-promoting index; UI: unsaturation index; ED: sum of eicosapentaenoic acid and docosahexaenoic acid; TFA: trans fatty acids.

**Table 3:** Binding free energy of enalaprilat and 24 fatty acids with cACE.

Compounds	Sources	AutoDock Binding Free Energy, ∆G (kcal/mol)
Enalaprilat	Commercial drug	−14.15
Hexanoic acid	Rho	−8.22
Decanoic acid	Rho	−9.02
Dodecanoic acid	Rho	−9.72
*cis*-9 Tetradecenoic acid	Rho, Ano	−10.06
Tetradecanoic acid	Rho, Ano	−10.18
Pentadecanoic acid	Rho, Ano	−10.1
(9Z)-hexadec-9-enoic acid	Rho, Ano	−10.54
Hexadecanoic acid	Rho, Ano	−10.17
*cis*-10 Heptadecenoic acid	Rho, Ano	−10.5
Heptadecanoic acid	Rho, Ano	−10.34
*cis*-6,9,12-octadecatrienoic acid	Rho, Ano	−11.37
*cis*-9,12-octadecadienoic acid	Rho, Ano	−11.21
(E)-9-octadecenoic acid	Rho, Ano	−10.99
*cis*-9-octadecenoic acid	Rho, Ano	−10.8
Octadecanoic acid	Rho, Ano	−10.44
*cis*-5,8,11,14-eicosatetraenoic acid	Rho, Ano	−11.72 ^(3),^*
*cis*-5,8,11,14,17-eicosapentaenoic acid	Rho, Ano	−11.78 ^(2),^*
*cis*-8,11,14-eicosatrienoic acid	Rho, Ano	−11.53
*cis*-11,14-eicosadienoic acid	Rho, Ano	−11.04
*cis*-4,7,10,13,16,19-docosahexaenoic acid	Rho, Ano	−12.37 ^(1),^*
*cis*-13-docosenoic acid	Ano	−11.57
Docosanoic acid	Ano	−10.71
*cis*-15-tetracosenoic acid	Ano	−11.23
Tetracosanoic acid	Ano	−11.28

Rho; Rhopalodia sp. AARL D020 and Ano; Anomoeoneis sp. AARL D039. * (1), (2), (3) are the binding energies sequence of the top three best fatty acids.

## Data Availability

Data are contained within the article.

## References

[1] Maneechote W., Cheirsilp B., Srinuanpan S., Pathom-aree W. (2021). Optimizing physicochemical factors for two-stage cultivation of newly isolated oleaginous microalgae from local lake as promising sources of pigments, PUFAs and biodiesel feedstocks. Bioresour. Technol. Rep..

[2] Lomakool S., Ruangrit K., Jeerapan I., Tragoolpua Y., Pumas C., Srinuanpan S., Pekkoh J., Duangjan K. (2021). Biological activities and phytochemicals profiling of different cyanobacterial and microalgal biomass. Biomass Convers. Biorefinery.

[3] Govindan N., Maniam G.P., Sulaiman A.Z., Ajit A., Chatsungnoen T., Chisti Y. (2021). Production of renewable lipids by the diatom *Amphora copulate*. Fermentation.

[4] Di Dato V., Di Costanzo F., Barbarinaldi R., Perna A., Ianora A., Romano G. (2019). Unveiling the presence of biosynthetic pathways for bioactive compounds in the *Thalassiosira rotula* transcriptome. Sci. Rep..

[5] Wang J.K., Seibert M. (2017). Prospects for commercial production of diatoms. Biotechnol. Biofuels.

[6] Conde T.A., Neves B.F., Couto D., Melo T., Neves B., Costa M., Silva J., Domingues M.R. (2021). Microalgae as sustainable bio-factories of healthy lipids: Evaluating fatty acid content and antioxidant activity. Mar. Drugs.

[7] Das U.N. (2004). Long-chain polyunsaturated fatty acids interact with nitric oxide, superoxide anion, and transforming growth factor-β to prevent human essential hypertension. Eur. J. Clin. Nutr..

[8] Sergeant S., Rahbar E., Chilton F.H. (2016). Gamma-linolenic acid, dihommo-gamma linolenic, eicosanoids and inflammatory processes. Eur. J. Pharmacol..

[9] Chen J., Liu H. (2020). Nutritional indices for assessing fatty acids: A mini-review. Int. J. Mol. Sci..

[10] Araujo P., Truzzi C., Belghit I., Antonucci M. (2021). The impact of seawater warming on fatty acid composition and nutritional quality indices of *Trematomus bernacchii* from the Antarctic region. Food Chem..

[11] Kheeree N., Sangtanoo P., Srimongkol P., Saisavoey T., Reamtong O., Choowongkomon K., Karnchanatat A. (2020). ACE inhibitory peptides derived from de-fatted lemon basil seeds: Optimization, purification, identification, structure–activity relationship and molecular docking analysis. Food Funct..

[12] Mirzaei M., Mirdamadi S., Safavi M. (2020). Structural analysis of ACE-inhibitory peptide (VL-9) derived from *Kluyveromyces marxianus* protein hydrolysate. J. Mol. Struct..

[13] Vilakazi H., Olasehinde T.A., Olaniran A.O. (2021). Chemical characterization, antiproliferative and antioxidant activities of polyunsaturated fatty acid-rich extracts from *Chlorella* sp. S14. Molecules.

[14] Verspreet J., Soetemans L., Gargan C., Hayes M., Bastiaens L. (2021). Nutritional Profiling and preliminary bioactivity screening of five micro-algae strains cultivated in Northwest Europe. Foods.

[15] Ruangrit K., Chaipoot S., Phongphisutthinant R., Duangjan K., Phinyo K., Jeerapan I., Pekkoh J., Srinuanpan S. (2021). A successful biorefinery approach of macroalgal biomass as a promising sustainable source to produce bioactive nutraceutical and biodiesel. Biomass Convers. Biorefinery.

[16] Pekkoh J., Ruangrit K., Pumas C., Duangjan K., Chaipoot S., Phongphisutthinant R., Jeerapan I., Sawangrat K., Pathom-aree W., Srinuanpan S. (2021). Transforming microalgal *Chlorella* biomass into cosmetically and nutraceutically protein hydrolysates using high-efficiency enzymatic hydrolysis approach. Biomass Convers. Biorefinery.

[17] Caballero J. (2020). Considerations for docking of selective angiotensin-converting enzyme inhibitors. Molecules.

[18] Bernstein K.E., Shen X.Z., Gonzalez-Villalobos R.A., Billet S., Okwan-Duodu D., Ong F.S., Fuchs S. (2011). Different in vivo functions of the two catalytic domains of angiotensin-converting enzyme (ACE). Curr. Opin. Pharmacol..

[19] Cozier G.E., Arendse L.B., Schwager S.L., Sturrock E.D., Acharya K.R. (2018). Molecular basis for multiple omapatrilat binding sites within the ACE C-domain: Implications for drug design. J. Med. Chem..

[20] Onufriev A.V., Alexov E. (2013). Protonation and pK changes in protein–ligand binding. Q. Rev. Biophys..

[21] Frisch M.J., Trucks G.W., Schlegel H.B., Scuseria G.E., Robb M.A., Cheeseman J.R. (2009). Gaussian 09, Revision B. 01.

[22] Fields F.J., Kociolek J.P. (2015). An evolutionary perspective on selecting high-lipid-content diatoms (Bacillariophyta). J. Appl. Phycol..

[23] Banskota A.H., Sperker S., Stefanova R., McGinn P.J., O’Leary S.J. (2019). Antioxidant properties and lipid composition of selected microalgae. J. Appl. Phycol..

[24] Lordan R., Tsoupras A., Zabetakis I. (2017). Phospholipids of animal and marine origin: Structure, function, and anti-inflammatory properties. Molecules.

[25] Yi Z., Xu M., Di X., Brynjolfsson S., Fu W. (2017). Exploring valuable lipids in diatoms. Front. Mar. Sci..

[26] Das U.N. (2008). Essential fatty acids and their metabolites could function as endogenous HMG-CoA reductase and ACE enzyme inhibitors, anti-arrhythmic, anti-hypertensive, anti-atherosclerotic, anti-inflammatory, cytoprotective, and cardioprotective molecules. Lipids Health Dis..

[27] Blasio M., Balzano S. (2021). Fatty acids derivatives from eukaryotic microalgae, pathways and potential applications. Front. Microbiol..

[28] Jusoh M., Loh S.H., Chuah T.S., Aziz A., San Cha T. (2015). Indole-3-acetic acid (IAA) induced changes in oil content, fatty acid profiles and expression of four fatty acid biosynthetic genes in *Chlorella vulgaris* at early stationary growth phase. Phytochemistry.

[29] Aliyu M., Kano M.A., Abdullahi N., Kankara I.A., Ibrahim S.I., Muhammad Y.Y. (2017). Extraction, characterization and fatty acids profiles of *Nymphaea Lotus* and *Nymphaea Pubescens* seed oils. Biosci. Biotechnol. Res. Asia.

[30] Yurchenko S., Sats A., Tatar V., Kaart T., Mootse H., Jõudu I. (2018). Fatty acid profile of milk from Saanen and Swedish Landrace goats. Food Chem..

[31] Matos Â.P., Feller R., Moecke E.H.S., de Oliveira J.V., Junior A.F., Derner R.B., Sant’Anna E.S. (2016). Chemical characterization of six microalgae with potential utility for food application. J. Am. Oil Chem. Soc..

[32] Rincón-Cervera M.Á., González-Barriga V., Romero J., Rojas R., López-Arana S. (2020). Quantification and distribution of omega-3 fatty acids in South Pacific fish and shellfish species. Foods.

[33] Buono S., Langellotti A.L., Martello A., Rinna F., Fogliano V. (2014). Functional ingredients from microalgae. Food Funct..

[34] EFSA (European Food Safety Authority) (2014). Drivers of Emerging Risks and Their Interactions in the Domain of Biological Risks to Animal, Plant and Public Health: A Pilot Study.

[35] Raff M., Tholstrup T., Sejrsen K., Straarup E.M., Wiinberg N. (2006). Diets rich in conjugated linoleic acid and vaccenic acid have no effect on blood pressure and isobaric arterial elasticity in healthy young men. J. Nutr..

[36] da Costa E., Melo T., Reis M., Domingues P., Calado R., Abreu M.H., Domingues M.R. (2021). Polar lipids composition, antioxidant and anti-inflammatory activities of the atlantic red seaweed *Grateloupia turuturu*. Mar. Drugs.

[37] Shetty V., Mokashi K., Sibi G. (2015). Variations among antioxidant profiles in lipid and phenolic extracts of microalgae from different growth medium. J. Fish. Aquat. Sci..

[38] Peraman M., Nachimuthu S. (2019). Identification and quantification of fucoxanthin in selected carotenoid-producing marine microalgae and evaluation for their chemotherapeutic potential. Pharmacogn. Mag..

[39] Kim Y.K., Kim Y.A., Shin S.B., Lee T.S., Yoon H.D. (2015). Angiotensin-I converting enzyme fatty acid inhibitory fractions from the Korean melania snail *Semisulcospira coreana*. Food Sci. Biotechnol..

